# Descriptive Epidemiology of Safety Events at an Academic Medical Center

**DOI:** 10.3390/ijerph17010353

**Published:** 2020-01-04

**Authors:** Alexandre R. Marra, Abdullah Algwizani, Mohammed Alzunitan, Theresa M. H. Brennan, Michael B. Edmond

**Affiliations:** 1 Department of Internal Medicine, University of Iowa Carver College of Medicine, Iowa City, IA 52242, USA; algwizani@gmail.com (A.A.); alzunitan@gmail.com (M.A.); theresa-brennan@uiowa.edu (T.M.H.B.); michael-edmond@uiowa.edu (M.B.E.); 2 Division of Medical Practice, Hospital Israelita Albert Einstein, 05652 São Paulo, Brazil; 3 Division of Infectious Diseases, Prince Mohammad Bin Abdulaziz Hospital, Riyadh 14214, Saudi Arabia; 4 Department of Infection Prevention and Control, King Abdulaziz Medical City, National Guard-Health Affairs, Riyadh 14611, Saudi Arabia

**Keywords:** patient safety, safety events, epidemiology

## Abstract

*Background*: Adverse safety events in healthcare are of great concern, and despite an increasing focus on the prevention of error and harm mitigation, the epidemiology of safety events remains incomplete. *Methods*: We performed an analysis of all reported safety events in an academic medical center using a voluntary incident reporting surveillance system for patient safety. Safety events were classified as: serious (reached the patient and resulted in moderate to severe harm or death); precursor (reached the patient and resulted in minimal or no detectable harm); and near miss (did not reach the patient). *Results*: During a three-year period, there were 31,817 events reported. Most of the safety events were precursor safety events (reached the patient and resulted in minimal harm or no detectable harm), corresponding to 77.3%. Near misses accounted for 10.8%, and unsafe conditions for 11.8%. The number of reported serious safety events was low, accounting for only 0.1% of all safety events. *Conclusions*: The reports analysis of these events should lead to a better understanding of risks in patient care and ways to mitigate it.

## 1. Introduction

Many healthcare organizations are engaged in improving their safety culture and transitioning to high reliability [1,2,3]. Reporting safety events improves the safety culture in hospitals [4,5], and by analyzing reported safety events, severe patient harm, including deaths, may be prevented [4]. Moreover, reporting and analysis of near miss events are valuable as these events represent the potential for future harm [3]. Within the United Kingdom of Great Britain and Northern Ireland, there are about 100,000 reports of patient-safety incidents from England and Wales every month [6]. The proportion of serious adverse events, although small (1 per 1000 admissions) cannot be negligible [7]. If this rate is applied to 34.7 million inpatients in the United States (US) [8], an estimated 35,000 patients per year could be seriously or permanently injured or could die during hospitalization due to an adverse event [7]. It is important to understand that it is necessary to influence decision-making on the implementation of quality safety systems and their processes in the US and international hospitals [1,2,3,4,5].

Healthcare Performance Improvement, a consulting firm, has developed a safety event classification that is currently used in some hospitals [9], and their methods can be used to detect and avoid safety events [2,3,10]. This classification is based on the degree of harm that results from a deviation from expected performance or standard of care [9].

Reporting safety events is important to the broad goal of error reduction [11]. Voluntary report systems are endorsed in the Institute of Medicine’s report on the errors in medical care [7,12]. Reporting will occur only if practitioners feel safe doing so and it becomes a culturally accepted activity when taking the measure of successful system changes, rather than events reported [11,13].

The purpose of this study is to delineate the descriptive epidemiology of safety events and patient outcomes in a hospital setting and to determine if these events are related to broad underlying themes to guide future actions that can impact patient safety in hospitals.

## 2. Materials and Methods 

This study was a quality improvement project that was approved by the Institutional Review Board (IRB) of the University of Iowa (IRB ID# 201601740). The University of Iowa Hospitals and Clinics (UIHC) is an academic tertiary-care hospital located in Iowa City, Iowa. During the study period, the hospital had 811 beds. Approximately 36,000 inpatients are admitted per year. The Patient Safety Program at UIHC focuses on patient safety, working in conjunction with the Joint Office for Compliance. This program started in 2007. All patients with reported safety events at the UIHC from 1 January 2014 to 31 December 2016 were retrospectively identified using an electronic safety event database (Patient Safety Net, Vizient, Chicago, IL, USA) that provides an online portal for passive surveillance. This incident reporting is a voluntary patient safety event reporting system, which relies on those involved in events to provide detailed information (Figure S1). Any healthcare worker (HCW) from our institution can submit an incident report via the electronic system. All submitted reports are reviewed daily, and the hospital’s Safety Oversight Team (physicians, nurses, quality engineers, associate chief quality officers, and chief quality officer) meets three times per week to discuss selected cases and to identify systems problems and systems solutions.

The AHRQ (Agency for Healthcare Research and Quality) Common Format Harm Score v1.1 was assigned to each submitted report. Using the score (1 through 9), each safety event was classified as: a serious safety event (a deviation from generally accepted performance standards that reached the patient and resulted in moderate to severe harm or death); a precursor safety event (reached the patient and resulted in minimal harm or no detectable harm); or a near miss safety event (did not reach the patient) [9]. Harm scores 7 (permanent harm), 8 (severe permanent harm), and 9 (death) were reclassified as serious safety events. Harm scores 3 (no harm evident, physical or otherwise), 4 (emotional distress or inconvenience), 5 (additional treatment), and 6 (temporary harm) were reclassified as precursor safety events. Harm score 2 events were considered near miss safety events. Harm score 1 (unsafe condition) cases were not reclassified (Table 1). 

Data from the Patient Safety Net system were exported to an Excel file and included age, gender, location of the patient (ward vs. ICU), type of safety event (adverse reaction; anesthesia event; behavioral event; care coordination/communication; complication of surgery; complication of care; problems with equipment/devices; fall; food/nutrition; medical records/patient identification; medication-related; omission/errors in assessment, diagnosis, or monitoring; radiology test; transfusion), type of harm, and patient outcome. 

To determine the preventability of a serious safety event, we adopted the best practice-based assessment method [14].

### Statistical Analysis

A descriptive analysis was performed to define the proportion of patients with specific safety events and outcomes of interest (no harm or the level of harm, including death). Differences in proportions were compared using a Chi-square test or Fisher’s exact test when appropriate. Mean values were reported ±1 SD (standard deviation). All tests of significance were two-tailed. 

## 3. Results

Over the three years, there were 31,817 events reported. Of these, 85.9% (27,315) were patient events and 14.1% (4502) were unsafe conditions events (Table 2).

The total number of patient admissions was 33,429 in 2014, 34,740 in 2015, and 36,366 in 2016. Almost two-thirds (66.6%) of the patient events involved unique patients. The remainder represented multiple events per patient. The mean age was 46.2 ± 25.9 years; 3.1% occurred in patients < 1-year-old, 23.7% in patients 1–18 years old, and 23.6% in patients ≥ 65 years old. The gender distribution was 45.4% female and 54.6% male.

The majority (77.3%) of safety events were precursor safety events (reached the patient and resulted in minimal harm or no detectable harm, Table 3). Near misses accounted for 10.8%, unsafe conditions for 11.8%, and serious safety events (reached the patient and resulted in moderate to severe harm or death) accounted for 0.1%. The rate of reported serious safety events was 5.6/100,000 patient days or 3.5/10,000 admissions. Less than 5% of reported events were associated with any level of physical harm. Emotional distress or inconvenience occurred in 23.5%, and 9.8% required additional treatment without harm (Table 3). Medication errors were the most prevalent event type (20.3%), followed by laboratory events (14.5%) and problems related to the coordination of care or communication (9.3%).

With regards to medication safety events, the most prevalent had a wrong dose (17.1%), followed by a narcotics discrepancy (10.2%), prescription issues (4.1%), and a wrong dosing frequency (3.4%). The most prevalent laboratory safety event was specimen labeling (73.6%), followed by specimen collection problems (10.4%), specimen quality issues (5.4%), and transport of specimen (1.8%). 

With regards to the location of the reported safety events, 12.8% occurred in ICUs, 35% occurred in wards, 13.6% occurred in the operating room, 4.7% in the emergency department, and 3.4% occurred in the ambulatory setting.

Evaluating the harm score categories by each event type (Table 4), we found that medication errors accounted for nearly one-half of near misses (40.4%). Coordination and communication issues accounted for 27.2% of severe permanent harm cases and falls accounted for 18.2%.

Of the 15 deaths reported in the study period, four were related to surgical or interventional procedures, which, on further review, were determined to be unavoidable complications in high-risk patients. One was related to an unhelmeted motorcycle crash with unsuccessful attempts at surgical and non-surgical procedures to secure the airway. Three cases were thought to be due to unusual adverse medication reactions, two of which were unavoidable, while the third was potentially avoidable (ciprofloxacin and warfarin interaction with close coagulation monitoring). In these cases, the Naranjo Adverse Drug Reaction Probability Scale score was in the possible range. Two cases were related to unavoidable fall-related injuries that occurred despite the implementation of universal fall precautions. Two cases involved self-harm in psychiatric patients (one inpatient, one outpatient), and the three remaining cases were thought to be due to the severity of their medical illnesses (pulmonary embolus associated with a vascular access device, severe H1N1 influenza pneumonia, and refractory normal pressure hydrocephalus).

The majority of falls (62.3%) resulted in no harm, as did laboratory events (50.7%) and surgical complications (45.1%). A significant proportion of equipment-related events (40.1%) and medication errors (38.7%) also caused no harm. Emotional distress was the most common harm associated with transfusion events (61.6%) and radiology events (40.3%) (Table 5).

Specific hospital settings were also analyzed separately. In the psychiatric units, which accounted for 1.5% of the patient safety events (*n* = 471), the most common reports were behavioral events (36%), falls (15.7%) and medication-related issues (10.4%). In the operating room (*n* = 4319), the principal safety events were issues related to the surgery (18.3%), care coordination/communication (8.8%), medication-related issues (8.8%), and transfusions (6.5%). In the emergency department (*n* = 1498), the most common patient safety events were related to laboratory tests (23%), care coordination/communication (16.3%), and transfusions (9.4%).

There was a statistically significant decrease in the number of serious safety events between 2014 and 2015 (15 vs. 7, OR 2.57 95% CI 1.05–6.30, *p* = 0.03), despite a 20% increase in the overall number of safety events reported. However, there was no statistically significant difference between 2014 and 2016 (15 vs. 15, OR 1.37 95% CI 0.67–2.81, *p* = 0.38), despite a 27% increase in the overall number of events reported. In addition, the proportion of safety events that were serious in ICU patients (0.2%, 9/4088) was significantly higher than in non-ICU patients (0.1%, 28/27,729; OR 2.18, 95% CI 1.03–4.63, *p* = 0.037).

## 4. Discussion

This study showed that the number of reported voluntarily serious safety events at a tertiary medical center was low, accounting for only 0.1% of all safety events. We hypothesized that we would find a higher proportion of serious safety events in ICUs than in non-ICU wards, given that intensive care patients are critically ill and undergo many invasive procedures [15], and this was confirmed by our study. Other studies have also demonstrated a higher number of adverse events in ICU patients [16] via medical record review [17,18], while our study analyzed safety events reported by HCWs through a passive surveillance system. A study conducted in 26 acute care hospitals throughout the United States, that also voluntarily reported safety events (92,527 reports), showed events that reached a patient made up the majority of reports, of which two-thirds caused no harm to the patient and slightly over 1% resulted in permanent or life-threatening harm or death [7]. A recent systematic review showed that more than one-third of systematic reviews did not fully report the outcome of the adverse events [19]. This is why we decided to publish our data and encourage other hospitals to report adverse events and promote research to mitigate their risks. 

From our analysis, it is important to note that the majority of our reported safety events were medication-related. Medication safety events are common, but often preventable, and impose substantial costs on the healthcare system [18]. Fortunately, nearly 40% of medication safety events were near misses. Of the three reported severe safety events that were medication-related, two were not preventable, while the third was possibly preventable. We adopted a practice assessment to evaluate the preventability of a severe safety event, due to the complexities and variations of these events in clinical practice [14]. Using the same method, a recent study showed that more than half (64%) of adverse drug events are probably or definitely preventable [20]. A published meta-analysis estimating the percentage of patients with preventable adverse drug reactions and the preventability of adverse drug reactions in adult outpatients and inpatients showed that among adult outpatients, 2% had preventable adverse drug reactions and 52% of adverse drug reactions were preventable; among inpatients, 1.6% had preventable adverse drug reactions and 45% of adverse drug reactions were preventable [21]. Of note, our hospital has used a bar-coding system for medication administration since 2009. A recent study showed a reduction of 43.5% in reported medication administration errors after implementing bar-code medication administration technology in the inpatient setting, resulting in a reduction of 55.4% in actual patient harm events [22]. 

Care coordination and communication issues were one of the most prevalent safety event types, and our descriptive analysis showed that these were responsible for almost 40% of reported events resulting in severe permanent harms. Although not all errors may be preventable, it is important to improve healthcare worker performance concerning cognitive and behavioral issues that impact communication [23]. A qualitative study highlighted that formal and informal communication is important in supporting patient care [24], but a recent integrative review did not find an intervention that is most effective for improving nurse–physician communication [25]. 

This study has several limitations. The main limitation was that the data were obtained through a passive surveillance system, where the reporting was voluntary, particularly since it is known that errors are underreported worldwide [26,27]. The advantages of voluntary event reporting systems include their relative acceptability and the involvement of frontline personnel in identifying safety hazards for the institutions. Voluntary event reporting systems are generally confidential and can also be anonymous: Neither the patient nor the reporter can be identified [8,28]. Another concern is that near miss safety events may be even more underreported, while serious safety errors are more likely to be reported [1,9,29]. What contributes to the high number of reported safety events (more than 30,000 in the study period) is a system that is non-punitive and easy to use. We have reported a wide variety of different types and severities of safety events and have not only recorded the most serious events. Similar findings were reported for previous studies [6,7,30]. On the other hand, events reported were subjected to selection bias due to their voluntary nature [28,31]. A recent systematic review about the classification of patient safety incidents in primary care found 38 studies, and these covered 21 distinct systems for the classification of harm severity [6]. Out of these 21 existing approaches to the classification of harm in patient-safety incidents, only six of these approaches took psychological outcomes, described as emotional, mental, or psychological harm [6]. However, for some safety events, the impact of psychological stress may be greater than physical harm [32]. Lastly, the events reported in our study were not assessed for preventability.

Previous studies on medical errors have shown a high degree of compliance with reporting [3,33]. Although our surveillance system may have had limitations, the reasoning for this belief was more efficient and thus was ultimately more effective than actively searching for safety events in medical records. Nonetheless, we were not able to estimate the proportion of serious safety events that were not reported. Education on the importance of reporting safety events, most importantly the near misses, must be an ongoing process.

## 5. Conclusions

Safety events are still of great concern and require continued monitoring. The reporting and analysis of these events should lead to a better understanding of risks in patient care and ways to mitigate it. Although the number of reported serious safety events was low, this is an opportunity to disseminate knowledge and motivate other institutions to implement evidence-based best practices for improving the quality and safety of patient care. 

## Figures and Tables

**Table 1:** Harm score classification.

Class	Score	Harm
Serious safety event	9	Death
	8	Severe permanent harm
	7	Permanent harm
Precursor safety event	6	Temporary harm
	5	Additional treatment
	4	Emotional distress or inconvenience
	3	No harm evident, physical or otherwise
Near miss safety event	2	Did not reach the patient
Unsafe condition safety event	1	Unsafe condition

**Table 2:** Reported safety events.

	Total	2014	2015	2016
Hospital admissions	104,535	33,429	34,740	36,366
Patient-days	657,084	207,973	214,194	234,917
Safety events	31,817	8911	10,663	12,243
Patient-related safety events	27,315	7927	9499	9889
Patients with one patient event reported, *n* (%)	18,202 (66.6)	5981 (75.5)	6663 (70.1)	5558 (56.2)
Patients with ≥2 patient events reported, *n* (%)	9113 (33.4)	1946 (24.5)	2836 (29.9)	4331 (43.8)
Unsafe condition events reported	4502	984	1164	2354
Serious safety events (SSEs)	37	15	7	15
Proportion of patient events that were SSE (%)	0.14	0.19	0.07	0.15
Rate of SSE (per 100,000 patient-days)	5.6	7.2	3.3	6.4

**Table 3:** Descriptive epidemiology of safety events.

Demographic Data	Total (*n* = 31,817)	2014 (8911)	2015 (10,633)	2016 (12,243)
Age (mean ± SD) *	46.6 ± 25.9	46.8 ± 26.2	46.8 ± 26.1	46.2 ± 25.6
Female gender	45.4	46.2	44.8	48.3
Location:	%	%	%	%
Intensive Care Unit	12.8	12.1	11.5	14.6
Non-Intensive Care Unit	87.2	87.9	88.5	85.4
Total	100	100	100	100
Harm score:	%	%	%	%
Unsafe condition	11.8	11.0	10.9	13.1
Near miss	10.8	9.7	9.4	12.9
No harm evident, physical or otherwise	39.2	42.4	42.4	34.1
Emotional distress or inconvenience	23.5	22.2	24.3	23.7
Additional treatment	9.8	9.6	9.9	9.8
Temporary harm	4.8	5.0	3.1	6.3
Permanent harm	<0.1	0.1	<0.1	<0.1
Severe permanent harm	<0.1	0.1	<0.1	<0.1
Death	0.1	<0.1	<0.1	<0.1
Total	100	100	100	100
Event type:	%	%	%	%
Medication	20.3	21.2	18.5	20.1
Laboratory	14.5	16.9	14.7	12.6
Coordination/communication	9.3	9.5	10.7	7.9
Equipment	7.8	8.0	7.9	7.6
Fall	7.3	6.9	7.1	7.8
Complication of care (surgical)	4.6	5.4	5.0	4.1
Transfusion	4.5	4.4	4.9	4.2
Radiology	3.1	4.1	3.0	3.1
Complication of care (non-surgical)	3.3	3.4	3.6	2.5
Others	25.2	20.2	24.6	30.1
Total	100	100	100	100

* Standard deviation (SD).

**Table 4:** Harm score and event type (percentage within harm score).

	Harm Score (*N* = 31,817)								
Event type	Unsafe Condition *N* = 3750 %	Near Miss *N* = 3439 %	No Harm Evident * N = 12,477 %	Emotional Distress ** N = 7469 %	Additional Treatment *N* = 3105 %	Temporary Harm *N* = 1540 %	Permanent Harm *N* = 10 %	Severe Permanent Harm *N* = 11 %	Death *N* = 16 %
Medication (*n* = 6465)	30.6	40.4	20.0	9.3	19.7	7.7	0.0	9.1	18.7
Laboratory (*n* = 4617)	1.7	7.7	18.8	24.3	3.7	1.0	0.0	0.0	0.0
Coordination/ communication (*n* = 2942)	8.6	7.4	8.0	14.9	6.5	3.5	0.0	27.2	12.5
Equipment (*n* = 2486)	17.2	11.8	8.0	3.1	5.6	1.8	0.0	0.0	0.0
Fall (*n* = 2336)	0.3	0.9	11.7	5.2	10.1	8.3	10.0	18.2	12.5
Complication of care (surgical) (*n* = 1464)	4.0	4.5	5.3	2.3	5.9	8.9	40.0	9.1	18.8
Transfusion (*n* = 1425)	0.6	1.0	3.7	11.8	0.7	0.3	0.0	0.0	0.0
Complication of care (non-surgical) (*n* = 989)	0.6	0.7	1.8	3.3	12.3	5.7	10.0	9.1	25.0
Radiology (*n* = 1063)	0.5	1.7	2.4	5.7	6.6	3.4	10.0	9.1	0.0
Others (*n* = 8030)	35.9	23.9	20.3	20.1	28.9	59.4	30.0	18.2	12.5
Total	100	100	100	100	100	100	100	100	100

* No harm evident, physical or otherwise; ** Emotional distress or inconvenience.

**Table 5:** Harm score and event type (percentage within event type).

	Harm Score									
Event Type (*N* = 31,817)	Unsafe Condition *N* = 3750	Near Miss *N* = 3439	No Harm Evident * N = 12,477	Emotional Distress ** *N* = 7469	Additional Treatment *N* = 3105	Temporary Harm *N* = 1540	Permanent Harm *N* = 10	Severe Permanent Harm *N* = 11	Death *N* = 16	Total
Medication (*n* = 6465) %	17.8	21.5	38.7	10.7	9.4	1.8	0	<0.1	0.1	100
Laboratory (*n* = 4617) %	1.4	5.7	50.7	39.4	2.5	0.3	0	0	0	100
Coordination/ communication (*n* = 2942) %	10.9	8.7	33.8	37.7	6.9	1.8	0	0.1	0.1	100
Equipment (*n* = 2486) %	26	16.4	40.1	9.4	7	1.1	0	0	0	100
Fall (*n* = 2336) %	0.5	1.3	62.3	16.8	13.4	5.5	<0.1	0.1	0.1	100
Complication of care (surgical) (*n* = 1464) %	10.2	10.5	45.1	11.7	12.5	9.4	0.3	0.1	0.2	100
Transfusion (*n* = 1425) %	1.6	2.4	32.6	61.6	1.5	0.3	0	0	0	100
Complication of care (non-surgical) (*n* = 989) %	2.2	2.2	22.9	24.6	38.6	8.9	0.1	0.1	0.4	100
Radiology (*n* = 1063) %	1.7	5.5	28	40.3	19.3	5	0.1	0.1	0	100
Others (*n* = 8030) %	16.8	10.3	31.6	18.7	11.2	11.4	<0.1	<0.1	<0.1	100

* No harm evident, physical or otherwise; ** Emotional distress or inconvenience.

## Supplementary Materials

The following are available online at https://www.mdpi.com/1660-4601/17/1/353/s1, Figure S1: University of Iowa Hospitals and Clinics Safety Event Algorithm.
