# Supplementary material for: Descriptive Epidemiology of Safety Events at an Academic Medical Center
Source: Int J Environ Res Public Health. 2020 Jan 4;17(1):353. doi: 10.3390/ijerph17010353 (PMC6982027; doi:10.3390/ijerph17010353)
Supplement: Supplementary file 1 [file ijerph-17-00353-s001.pdf]

International Journal of
Environmental Research and Public Health

MDPI

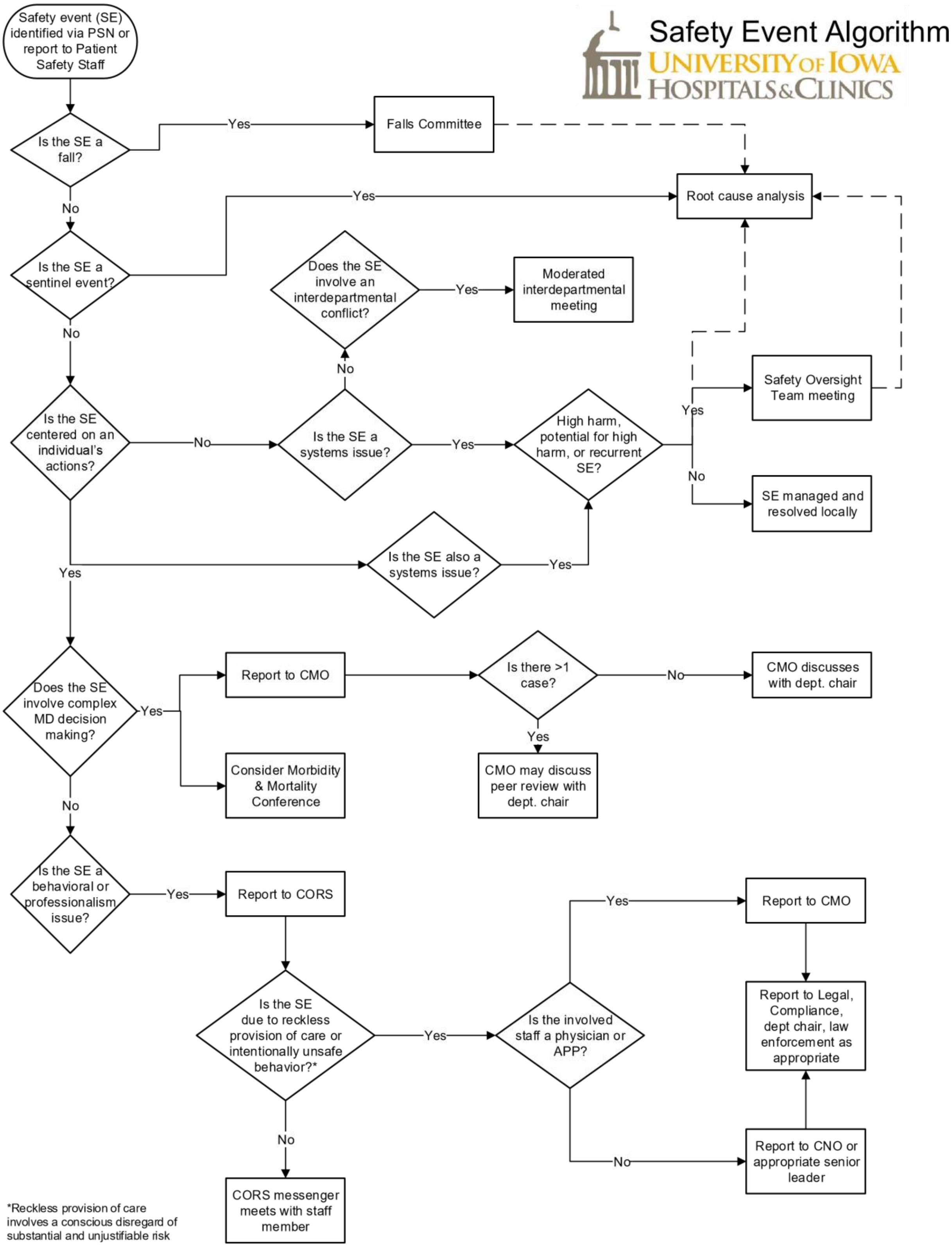

Figure S1. University of Iowa Hospitals and Clinics Safety Event Algorithm. SE = Safety Event; PSN = Patient Safety Net; CMO = Chief Medical Officer; MD = Medical Doctor; CORS = Co-worker Observations Reporting System; APP = Advanced Practice Provider; CNO = Chief Nursing Officer.
